# Insight into the characteristics, removal, and toxicity of effluent organic matter from a pharmaceutical wastewater treatment plant during catalytic ozonation

**DOI:** 10.1038/s41598-018-27921-0

**Published:** 2018-06-25

**Authors:** Shuhan Wen, Lin Chen, Weiqi Li, Hongqiang Ren, Kan Li, Bing Wu, Haidong Hu, Ke Xu

**Affiliations:** State Key Laboratory of Pollution Control and Resource Reuse, School of the Environment, Nanjing University, N.O.163, Xianlin Avenue, Qixia District, Nanjing, 210023 Jiangsu PR China

## Abstract

Changes in the characteristics, removal efficiency, and toxicity of pharmaceutical effluent organic matter (EfOM) after catalytic ozonation were investigated in this study. After a 90-min treatment with a catalytic ozonation process (COP) in the presence of MnO_2_ ceramsite, the total organic carbon (TOC), UV_254_, colority, protein, and humic acid removal rates were 13.24%, 60.83%, 85.42%, 29.36% and 74.19%, respectively. The polysaccharide content increased by 12.73 mg/L during the COP for reaction times between 0 and ~50 min and decreased by 6.97 mg/L between 50 and ~90 min. Furthermore, 64.44% of the total colority was detected in the hydrophobic organic matter (HOM) fraction, and after the COP, and 88.69% of the colority in the HOM was eliminated. Meanwhile, only 59.18% of the colority in the hydrophilic organic matter (HIM) fraction was removed. GC-MS analysis showed that 38 organic pollutant species were completely removed, 8 were partially removed, and 7 were generated. After 90 min of COP treatment, the pharmaceutical EfOM toxicity was effectively reduced based on the higher incubation and lower mortality rates.

## Introduction

With the rapid growth of pharmaceutical needs, large quantities of wastewater containing products, raw materials, solvents and detergents from complex manufacturing processes are generated^1^. Even conventional biological wastewater treatment facilities produce effluent organic matter (EfOM) with high chemical oxygen demand (COD), salinity, color, limited biodegradation, and toxicity^2,3^, which increase the potential risk to receiving waters and human health^4^. Therefore, further EfOM removal during pharmaceutical wastewater treatment is an urgent need.

Advanced chemical oxidation processes (AOPs) have attracted much attention for the advanced treatment of industrial wastewater, such as the base process of O_3_, H_2_O_2_, Peroxone, sulfate radical and photocatalytic^5–9^. Among these AOPs, the catalytic ozonation process (COP) has a strong ability to degrade refractory organic pollutants and effectively decolor water via hydroxyl radicals (·OH)^10,11^ and broad application potential for wastewater treatment. However, studies on the application of the COP for the advanced treatment of pharmaceutical industrial wastewater have been rarely reported.

For COPs, MnO_x_-based catalysts are promising catalysts with great advantages, such as high stability, low water solubility, environmental friendliness and ease of manufacture, and they have been widely studied and applied^12–14^. In addition, porous materials with large surface areas and abundant porous structures have been widely used as metal oxide carriers because of their good performance for increasing the active surface area and adsorption capacity^15,16^. Among these carriers, attapulgite (ATP) is a type of natural, hydrated magnesium silicate mineral with unique pore channels, a large surface area, a high adsorption capacity, and water-insolubility^17–19^.

Many studies have been performed with the COP to examine removal efficiencies in simulated organic pollution and actual wastewater^12–14^. Lei Zhao *et al*.^12^ prepared ceramic, honeycomb-supported manganese catalysts via an impregnation method in a solution of manganese salt and explored the effects of the main operating variables, such as the initial pH, reaction temperature and amount of catalyst, on the degradation efficiency of nitrobenzene. Although the ozonation/Mn-ceramic honeycomb system resulted in a removal rate of approximately 74% for nitrobenzene under the optimal experimental conditions, the quality of the background water was different from that of actual industrial wastewater. Chunmao Chen *et al*.^13^ treated heavy oil-refinery wastewater by an integrated ozone and activated carbon-supported manganese oxide method and observed a total organic carbon (TOC) reduction efficiency of 38% due to the catalytic effect. Fengxia Deng *et al*.^14^ increased the COD removal efficiency of refinery wastewater by approximately 30% using ozone and alumina-supported manganese and copper oxide catalysts compared with a single ozonation process. In summary, most studies have focused on the following characteristics: (1) new catalyst preparation and characterization of the structure and components, (2) organic pollutant degradation efficiency, (3) the influence of key operating parameters, (4) catalytic mechanism, and (5) catalyst reusability and stability. Thus far, systematic and detailed research on the changes in pharmaceutical EfOM during the COP is lacking.

In this study, ATP was used as a carrier and directly mixed with MnO_2_ particles to prepare the MnO_2_ ceramsite catalyst. The main aim of this study was to investigate the characteristics, removal, and toxicity of EfOM from a pharmaceutical wastewater treatment plant during a COP in the presence of MnO_2_ ceramsite to provide a theoretical basis for advanced treatments of pharmaceutical wastewater using catalytic ozonation technology. The effectiveness of the COP was measured by the UV_254_ and the TOC removal rate of the pharmaceutical wastewater. Second, the color degradation during the COP was explored via Fourier transform infrared (FT-IR) spectroscopy analysis, and the color degradation in the hydrophobicity/hydrophilicity of the EfOM. Then, the changes in the EfOM during the COP were observed by studying the changes in the soluble microbial products (SMPs, containing proteins, polysaccharides, and humic acid), molecular-weight distribution and hydrophobicity/hydrophilicity of the EfOM and performing gas chromatography-mass spectrometer (GC-MS) analyses. Finally, the toxicity of the pharmaceutical EfOM after the COP treatment was assessed to determine its potential risks, as mentioned earlier.

## Results and Discussion

### UV_254_ and TOC removal

**Figure 1:**
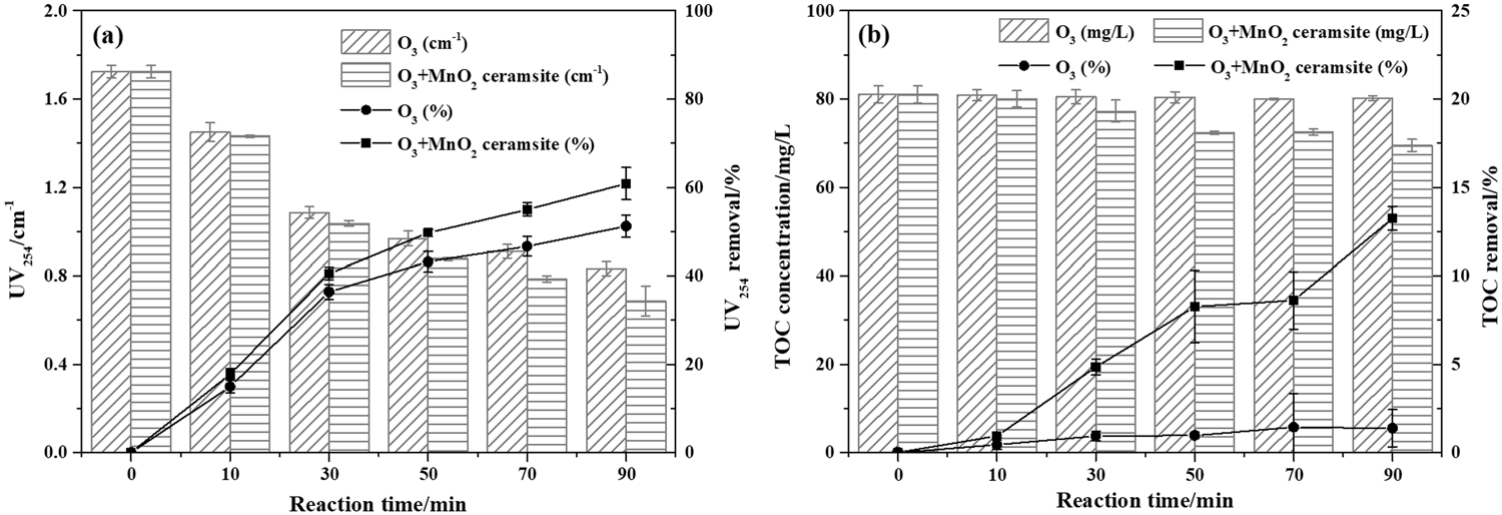
The concentrations and removal efficiencies of UV_254_ (**a**) and TOC (**b**) in the SOP and COP (experimental conditions: pH_0_, 3.11; catalyst dosage, 4.0 g/L; gas flow, 400 mL/min; inlet ozone concentration, 20.25 mg/L; reaction volume, 1.0 L; reaction time, 90 min).

UV_254_ represents the aromatic structure and organic pollutant double bond content^20^, and the organic pollutant content in pharmaceutical wastewater can be directly determined via the TOC value. The UV_254_ and TOC removal efficiencies in both the sole ozonation process (SOP) and COP are shown in Fig. 1. The results indicate that the SOP is almost ineffective for TOC removal, but it is quite effective in the destruction of aromatic compounds and double-bond systems. Under the experimental conditions, the UV_254_ and TOC removal efficiencies by the COP were 60.83% and 13.24%, respectively, which were 9.64% and 11.87% higher than those of the SOP. Thus, compared to the SOP, the COP can more completely break aromatic structures and double bonds, and some organic pollutants are mineralized during the COP. Furthermore, there is a strong correlation between the reduction of UV_254_ and the production of ·OH during ozonation^21^, which indicates that more ·OH is generated during the COP.

The degradation of UV_254_ during COP can be divided into two parts: the first one is a fast reaction period (0~30 min) with UV_254_ removal efficiency of 40.50%, and the second one is a slow reaction period (30~90 min) with UV_254_ removal efficiency of 20.33%. Previous studies^12,22^ have shown that MnO_x_-based catalysts can promote the initiation of hydroxyl radicals. Hence, during the COP, a decrease in the UV_254_ value is due to the ozone in the solution and the generation of hydroxyl radicals, which can destroy aromatic structures and double bonds and degrade macromolecular organic compounds into small organic molecules. Ozone can easily react with aromatic compounds with electron-donating groups, but it does not easily react with aromatic compounds with electron-withdrawing groups^23^. During the fast reaction period, the UV_254_ value attributed to aromatic compounds with electron-donating groups quickly decreases due to the dissolved ozone in solution. At the same time, ozone molecules accumulate in the wastewater and are converted to hydroxyl radicals which could react with both electron-donating groups and electron-withdrawing groups. During the slow reaction period, the hydroxyl radicals can react with aromatic compounds containing electron-withdrawing groups, resulting in a continuous decrease in the UV_254_ value. However, due that the production of hydroxyl radicals in SOP is smaller than COP, COP has a higher UV_254_ removal rate during the slow reaction period, and most fatty compounds with carbonyl and carboxylic acid groups which reacted with ozone would remain their structures till the end of treatment in SOP.

Ozone oxidation of organic matter is selective; i.e., ozone can more easily cleave benzene rings and double bonds to form small organic molecules rather than further degrade the small organic compounds to achieve complete mineralization^24–26^. Therefore, after an ozone treatment for 90 min, the TOC value of the pharmaceutical wastewater remained almost the same, and the TOC removal rate was approximately 1.37%, while the UV_254_ removal efficiency could reach 51.19%. However, upon the addition of MnO_2_ ceramsite, a higher concentration of ·OH (the study of ·OH generation could be seen in supporting information), which has no oxidation selectivity, was generated and improved the mineralization rate of the organic matter, resulting in a 13.24% removal rate. The slightly low value of TOC removal rate may be due to the presence of various inorganic anions in wastewater. Inorganic anions, such as chloride ions (Cl^−^), sulfate ions (SO_4_^2−^), bicarbonate ions (HCO_3_^−^) and phosphate ions (PO_4_^3−^), may have an effect on the degradation of organic matter in the COP^27–29^. There are three major anions in the bio-treated pharmaceutical wastewater (BTPW): Cl^−^, PO_4_^3−^ and NO_3_^−^. PO_4_^3−^, which is a stronger Lewis base than the water, can substitute for surface OH groups on the catalysts by adsorption on the metal oxide in water^30,31^, and is considered hydroxyl radical scavenger^32^. Cl^−^, which acts as a typical hydroxyl radical scavenger in aqueous solutions, can react with ozone and ·OH to generate less reactive chlorine species^29,33^. Nevertheless, Zhang *et al*.^34^ and Yuan *et al*.^27^ found that the introduction of Cl^−^ could promote the degradation of organics, which was caused by the complex formation between Cl^−^ and the catalyst and the presence of main oxidizing free radical was O_2_^−^ instead of ·OH. NO_3_^−^ has no significant effect on the removal of organic matter^29^.

### Decolorization performance

**Figure 2:**
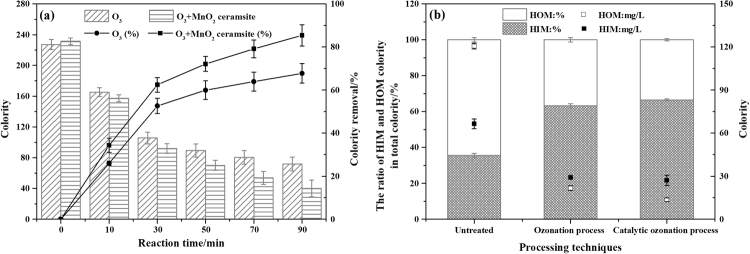
The colority concentration and removal efficiency in the SOP and COP (**a**) and the colority distribution in the untreated HIM and HOM fractions and the HIM and HOM fractions treated with the SOP and COP (**b**) (experimental conditions: pH_0_, 3.11; catalyst dosage, 4.0 g/L; gas flow, 400 mL/min; inlet ozone concentration, 20.25 mg/L; reaction volume, 1.0 L; reaction time, 90 min).

Although the pharmaceutical wastewater was previously treated by hydrolysis acidification, anaerobic-anoxic-oxic (A/A/O) and a moving-bed biofilm reactor (MBBR) in sequence, the water still had a very high colority of 224. An XAD-8 resin was used to separate the BTPW into hydrophilic (HIM) and hydrophobic (HOM) organic matters. Figure 2 shows the concentration and removal efficiency of the colority as well as the distribution of the colority in the HIM and HOM fractions during the SOP and COP. As shown in Fig. 2(b), most of the wastewater colority is concentrated in the HOM fraction, accounting for 64.44% of the total colority. Qi *et al*. stated that in wastewater treatment plant, the hydrophobic fraction contains more unsaturated structures, whereas the hydrophilic fraction contains more carbohydrates or O-alky groups^35^. Figure 2(a) indicates that both the SOP and COP were good at removing the colority. Similar to the degradation of UV_254_, the colority reaction process can also be divided into a fast reaction period (0~30 min) and a slow reaction period (30~90 min). After 90 min of the COP treatment, 85.42% of the colority was removed, which was 17.66% higher than the removal in the SOP. Additionally, colority of 107.02 was decreased by the COP in the HOM fraction, and the degradation rate was 88.69%, which was 6.67% higher than that of the SOP. However, only 39.39 of the colority was removed by the COP in the hydrophilic wastewater, i.e., a 59.18% degradation rate, which was still 2.98% higher than that of the SOP.

**Figure 3:**
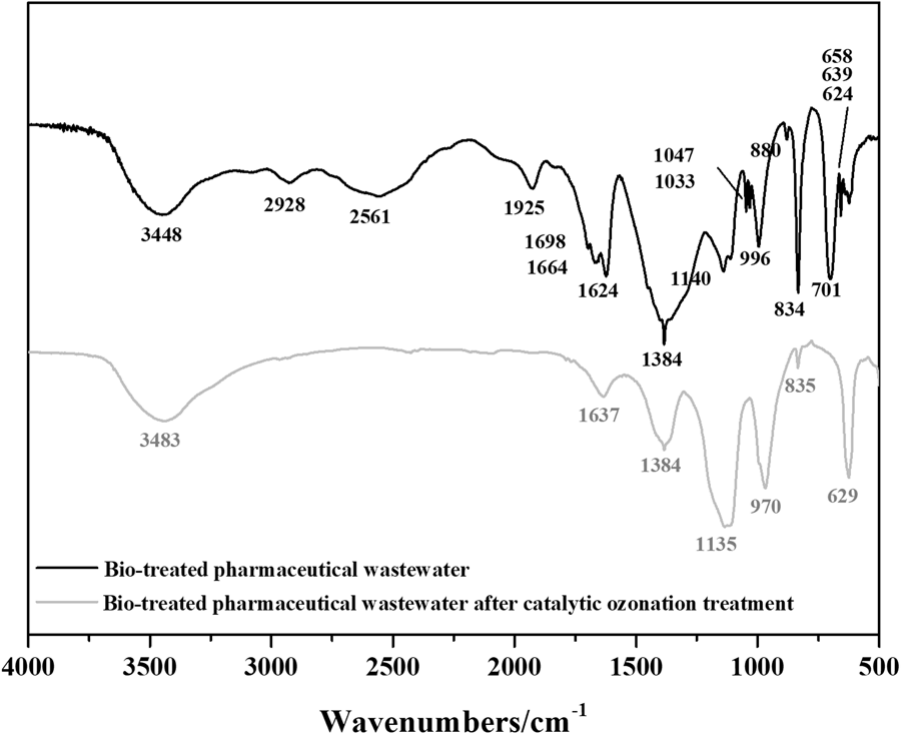
FT-IR spectra of dried samples derived from the BTPW before and after the COP.

The chromophoric and auxochromic functional groups of organic matter in BTPW could be analyzed by FT-IR spectroscopy (Fig. 3) in the range between 500 and 4000 cm^−1^. In BTPW, the band at 3348 cm^−1^ could be attributed to -NH_2_ or -OH^35,36^, which are important auxochromic groups, and this band remained after 90 min of the COP treatment. Furthermore, -OH is derived from the carboxyl, phenol and alcohol groups in the samples, and the presence of -OH and -NH_2_ contributes to the formation of colority. The peak at 2928 cm^−1^ and a low intense peak at 1440 cm^−1^ reflected C-H stretching of the cyclic ring^37,38^. These two peaks disappearing after COP possibly reflected pollutants containing cyclic ring, like 1,4-Dioxane and 1-Cyclohexene-1-carboxylic acid,4-(1,5-dimethyl-3-oxohexyl)-, methyl ester-, [R-(R*,R*)], had been degraded. The peak at 2561 cm^−1^ possibly reflect the asymmetric stretching vibration of –C=C-C=C- in the olefin structure. A peak at 1925 cm^−1^ due to out-of-plane vibration of =C-H in benzene ring^39^. The peak disappeared after the COP, which indicated that the structure of benzene ring was destroyed. The FT-IR spectra showed absorption peaks at 1698, 1664 and 1624 cm^−1^, which may be due to the stretching vibration of C=C and N=O^40^. After the COP, the peaks at 1698, 1664 and 1624 cm^−1^ significantly weakened to a single peak at 1637 cm^−1^, which resulted in a decrease in the colority and indicated of formed of C=O in primary amide groups^41,42^. The peak at 1384 cm^−1^ decreased after the COP, which indicated that a bond in -CH_3_ was broken. The peaks at 1047 cm^−1^, 1033 cm^−1^ and 996 cm^−1^ were probably caused by a high concentration of functional groups rich in oxygen with aliphatic structures^43^ or -C-O in alcohols and minerals^44^, and these bands disappeared after COP. The intense absorption peaks 880 cm^−1^ and 834 cm^−1^ might be due to the out-of-plane O-H bending of aromatics^45^. After COP, the weakening peak at 834 cm^−1^ and disappearance peak at 880 cm^−1^ might be due to the destruction of the aromatics. The bands at 624, 639, 658 and 701 cm^−1^ might be caused by the presence of carboxylate dimers, amines, and amides^46^. After COP, the bands formed at 1135 cm^−1^, 970 cm^−1^ and 629 cm^−1^, which might be due to the -C-O stretching vibration in the O-alky group or carbohydrate C-C^35^, the C-O-C vibration in ketals or hemiketals and the CNO vibrations in aliphatic nitro compounds^39^.

### Characteristics of EfOM during the COP

**Figure 4:**
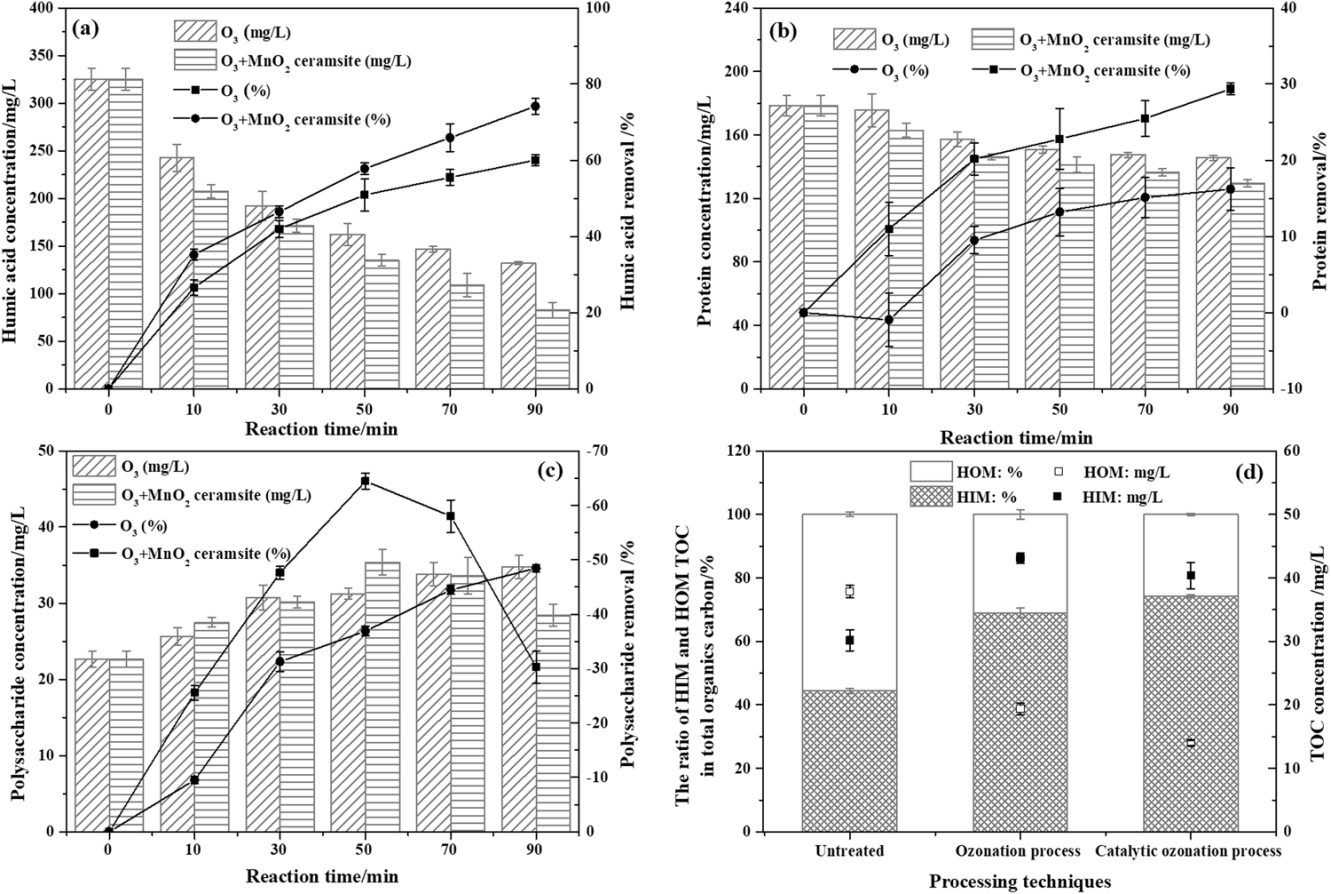
The concentrations and removal efficiencies of humic acid (**a**), protein (**b**) and polysaccharides (**c**) in the SOP and COP, and the distribution of TOC in the untreated HIM and HOM fractions and the HIM and HOM fractions treated with the SOP and COP (**d**) (experimental conditions: pH_0_, 3.11; catalyst dosage, 4.0 g/L; gas flow, 400 mL/min; inlet ozone concentration, 20.25 mg/L; reaction volume, 1.0 L; reaction time, 90 min).

In general, EfOM mainly contains natural organic matter (NOM), SMPs, and trace harmful chemicals, more specifically, EfOM includes proteins, polysaccharides, DNA, humic acid and other components^47^. Figure 4(a–c) shows the concentrations and removal efficiencies of humic acid, protein and polysaccharides for different processes. During the COP, the concentration of humic acid decreased from 325.36 mg/L to 82.73 mg/L, and the removal rate was 74.19%. The protein concentration was reduced by 49.11 mg/L, resulting in a removal rate of 29.36%. Similar trends were observed for the SOP: the removal rate of humic acid and protein was 60.02% and 16.22%, respectively. However, compared with those in the SOP, the removals of humic acid and protein increased by 12.17% and 13.14%, respectively, in the COP. In both the SOP and COP, the polysaccharide content significantly increased when the reaction time was 0~50 min, and the rate of increase in the polysaccharides was far greater in the COP than the SOP. When the reaction time was 50~90 min, the polysaccharide concentration began to decrease in the COP, while in the SOP, the polysaccharide content still increased at a lower rate. After a reaction time of 90 min, the polysaccharide content in the COP was 28.42 mg/L, which was 34.75 mg/L lower than that in the SOP.

Generally, in BTPW, the proteins and polysaccharides mainly consist of macromolecular organic polymers attached to microbial cell walls. Cytoplasm, which contains proteins and polysaccharides, can enter the solution after ozone and hydroxyl radicals directly or indirectly oxidize the cell wall and cell membrane during the COP, leading to an increase in the protein and polysaccharide concentrations. As more hydroxyl radicals are generated, the dissolution of intracellular material is accelerated. However, as the reaction proceeds, the release rate of polysaccharides from cells is less than the ozone and hydroxyl radical degradation rate. Therefore, in the COP, the polysaccharide production rate decreases for reaction times between 0 and 50 min, while the degradation rate increases for reaction times between 50 and 90 min.

The distribution of TOC in the HIM and HOM fractions is shown in Fig. 4(d). The wastewater contained 55.62% of HOM, 37.81 mg/L of TOC, and 44.28% of HIM, and this composition is similar to that observed in reverse osmosis concentrates^48^ and municipal wastewater^49^. The hydrophilicity of the EfOM was enhanced by the COP treatment to 74.22%. Similar results were reported by Orta de Velasquez *et al*.^50^, Hu *et al*.^51^ and Weng *et al*.^48^. The conversion of HOM to HIM is likely a result of organic matter with aromatic rings and alkyl groups, such as humic acid, reacting with O_3_ and ·OH to generate hydrophilic reaction products, such as carboxylic acids^52^. In the SOP and COP, the TOC values of HOM increase by 12.94 mg/L and 10.19 mg/L, respectively, while the TOC values of HIM decrease by 18.46 mg/L and 23.80 mg/L, respectively, indicating that more hydrophobic organic matter is converted to hydrophilic organic matter and can be completely mineralized in the COP than the SOP.

**Figure 5:**
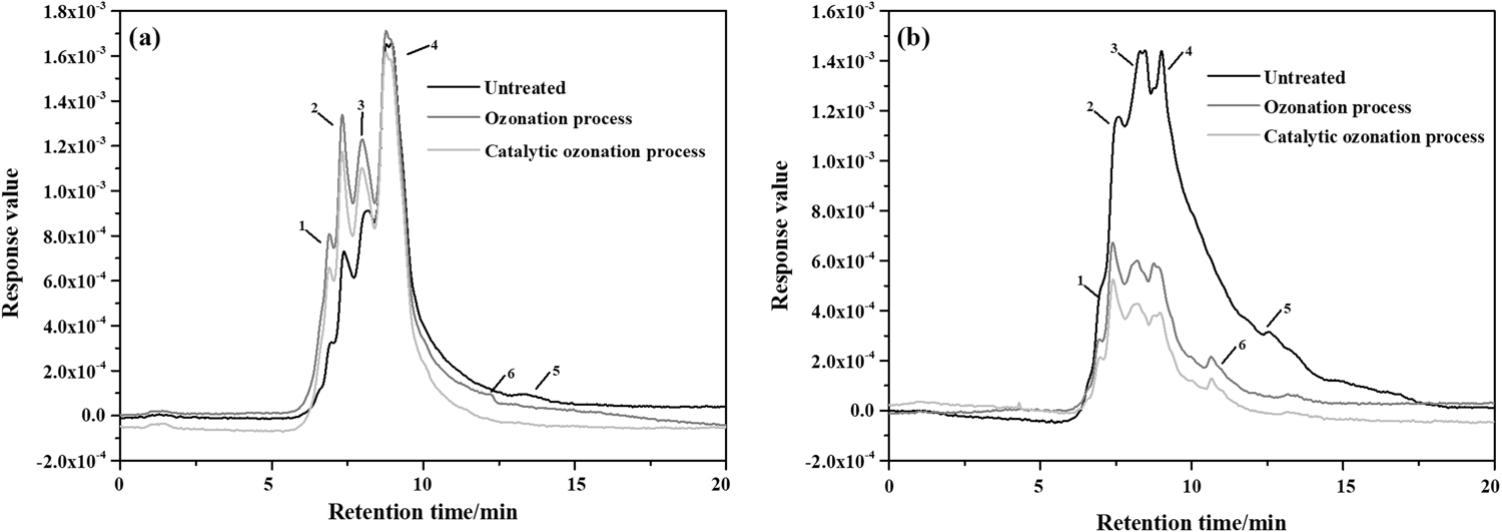
Changes in the molecular weight of untreated HIM and HOM in the BTPW and HIM and HOM fractions treated with the SOP and COP; (**a**) HIM and (**b**) HOM.

Figure 5 reflects the changes in the molecular weights of the HIM and HOM fractions in the BTPW during the different treatment processes (the details of retention time could be seen in supporting information). For the untreated HIM, the weight spectrum contains five peaks, and the 5th peak disappears after the COP, indicating that the hydrophilic small molecule organic substance was degraded. The areas of peaks 1–4 in Fig. 5(a), which have retention times similar to those of peaks 1–4 in Fig. 5(b), significantly increase, which is probably due to the conversion of HOM during the COP. The area of peak 4 is almost unchanged in Fig. 5(a), but the area significantly decreases in Fig. 5(b), indicating that the organic matter with a molecular weight of 700–1000 Da is degraded by the COP. For the HOM, the generation of peak 6 after the SOP and COP may result from the oxidation of larger molecular-weight HOM into smaller molecular organic compounds. The molecular-weight distribution of the ozone-treated wastewater is similar to that of the catalytic ozonation-treated samples. In both Fig. 5(a,b), the total peak area for the COP was lower than that for the SOP, indicating that the COP can promote the conversion of more HOM to HIM and increase the degradation rate of HIM.

A total of 45 organic pollutant species were detected by GC-MS in BTPW; after the COP, the content of 8 species decreased, and 38 species were completely removed. Seven new species were generated. The sum of the peak areas for all the organic compounds decreased by 64.27% for the COP, which was 10.37% less than that of the SOP and indicated that organic pollutant species are effectively degraded during the COP. The main organic pollutant species detected with relative peak areas >2% before and after the COP are listed in Table 1. The response value of macromolecular HOM decreased after the COP, which was probably due to the degradation of tetratetracontane, pentacosane, tetracosane and other macromolecule organic matter. In addition, the generation of new acids, esters and ketones, e.g., z-8-methyl-9-tetradecenoic acid, 1,2-benzenedicarboxylic acid, butyl 8-methylnonyl ester and 7,9-di-tert-butyl-1-oxaspiro(4,5)deca-6,9-diene-2,8-dione, enhanced the EfOM hydrophilicity. The reduction in the UV_254_ and colority values was likely a result of the partial degradation of organic compounds with double bonds and benzene rings, such as 2,4-di-tert-butylphenol-,[1,1′-biphenyl]-2,3′-diol,3,4′,5,6′-tetrakis(1,1-dimethylethyl) and 17-pentatriacontene. In summary, the COP can efficiently remove organic pollutants.

**Table 1:** Main organic pollutant species detected by GC-MS before and after the COP.

Organic compounds	Chemical formula	Raw sample		Catalytic ozonation	
Full name		RPA^a^	PA^b^	RPA	PA
2,4-Di-tert-butylphenol-	C_14_H_22_O	16.97	1.46E + 06	42.96	1.32E + 06
Tert-butyldimethylsilanol	C_6_H_16_OSi	10.57	9.11E + 05	ND^c^	ND
1,4-Dioxane	C_4_H_8_O_2_	8.56	7.38E + 05	6.55	2.02E + 05
Diisooctyl phthalate	C_24_H_38_O_4_	5.93	5.11E + 05	8.29	2.55E + 05
Octadecane,3-ethyl-5-(2-ethylbutyl)	C_26_H_54_	5.35	4.61E + 05	ND	ND
1,2-Benzenedicarboxylic acid, bis(2-methylpropyl) ester	C_16_H_22_O_4_	3.46	2.98E + 05	ND	ND
Tetratetracontane	C_44_H_90_	3.42	2.95E + 05	ND	ND
Heptacosane	C_27_H_56_	3.31	2.85E + 05	3.31	1.02E + 05
Benzenamine,N,N-dimethyl-4-[2-(4-quinolinyl)ethenyl]	C_19_H_18_N_2_	3.22	2.77E + 05	ND	ND
Pentacosane	C_25_H_52_	2.94	2.53E + 05	ND	ND
Tetracosane	C_24_H_50_	2.56	2.21E + 05	ND	ND
17-Pentatriacontene	C_35_H_70_	2.54	2.19E + 05	4.15	1.28E + 05
[1,1′-Biphenyl]-2,3′-diol,3,4′,5,6′-tetrakis(1,1-dimethylethyl)	C_28_H_42_O_2_	2.28	1.96E + 05	3.74	1.15E + 05
1,2-Benzenedicarboxylic acid, butyl-8-methylnonyl ester	C_22_H_34_O_4_	ND	ND	9.56	2.95E + 05
Z-8-Methyl-9-tetradecenoic acid	C_15_H_28_O_2_	ND	ND	5.69	1.75E + 05
Octadecane	C_18_H_38_	ND	ND	3.22	9.91E + 04
7,9-Di-tert-butyl-1-oxaspiro(4,5)deca-6,9-diene-2,8-dione	C_17_H_24_O_3_	ND	ND	2.83	8.71E + 04
Hexadecane	C_16_H_34_	ND	ND	2.36	7.26E + 04
2-Methylelcosane	C_21_H_44_	ND	ND	2.29	7.05E + 04
Octadecanal	C_18_H_36_O	1.19	102903	2.20	6.78E + 04

^a^Peak area.

^b^Relative peak area (%).

^c^ND, not detected.

### Toxicity assessment

**Figure 6:**
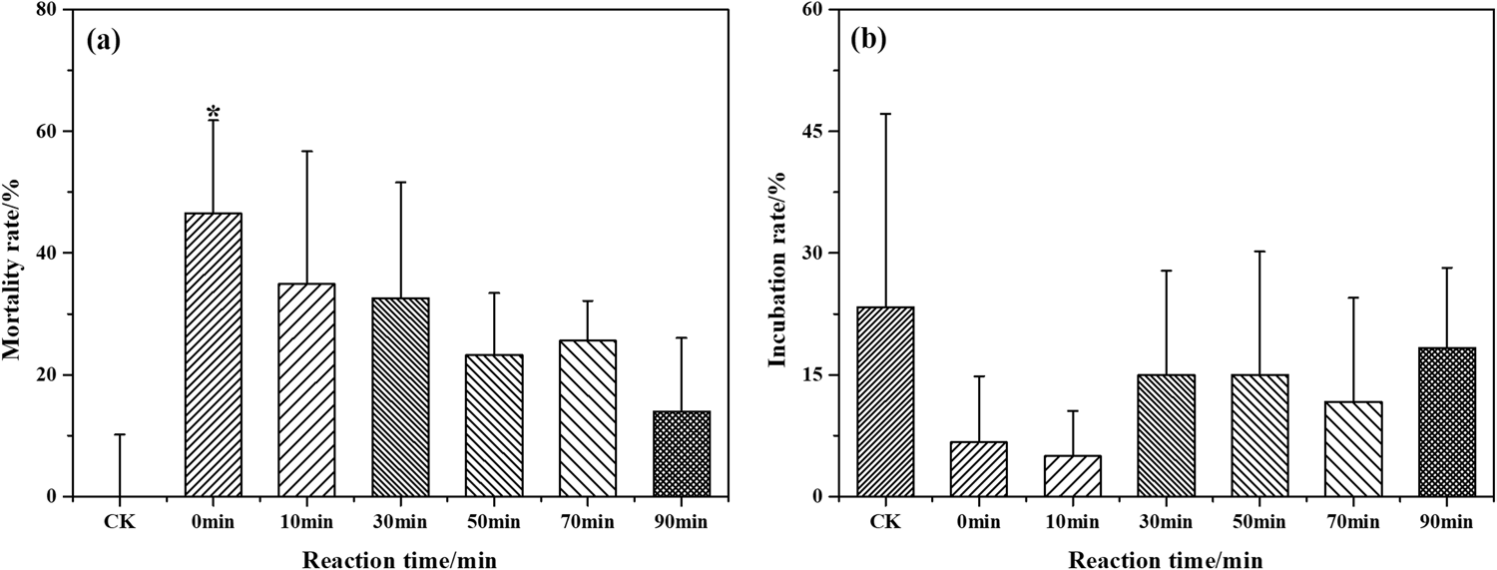
The effect of the COP reaction time on the zebrafish embryo 24 hpf mortality rate (**a**) and 48 hpf incubation rate (**b**).

Although the colority and organic matter were removed from the BTPW by the COP treatment after a reaction time of 90 min, the toxic effects of wastewater on the environment and human health cannot be ignored. A study explored the acute toxicity of reverse osmosis concentrates^48^ and showed that ozonation can increase the toxicity of concentrates for zebrafish and cause a higher mortality rate. The 24 hpf mortality and 48 hpf incubation rate of zebrafish embryos were used as acute toxicity assessment indicators in this study, and the results are shown in Fig. 6. Significant differences (p < 0.05) in the 24 hpf mortality of the zebrafish embryos existed between the control group and the untreated BTPW. The BTPW had high biological toxicity with a high mortality rate of 46.51% and a low incubation rate of 6.67%. The mortality rate gradually decreased and the incubation rate increased as the COP reaction time increased. At the end of the reaction, the mortality and incubation rates were 13.95% and 18.33%, respectively, which were not significantly different from the control group and indicated that the COP could effectively reduce the toxicity of BTPW. The results show that the COP can effectively eliminate the BTPW toxicity, and this technique can be used as a reliable method for the detoxification of pharmaceutical wastewater.

## Conclusions

Experiments were performed to study the removal rates and toxicity of BTPW during the COP in the presence of MnO_2_ ceramsite. The following conclusions were obtained:

1. The COP has a good performance for the removal of TOC and UV_254_ with 60.83% and 13.24% removal rates, respectively. Furthermore, the COP had a high efficiency for the removal of proteins, polysaccharides and humic acid. In addition, the COP can promote the conversion of HOM to HIM and increase the degradation rate of HIM.
2. The COP removed 85.42% of the colority by destroying –C=C-C=C-, C=O, N=O, and C=C bonds. Furthermore, most of the colority in the HOM was eliminated with an 88.69% degradation rate, whereas only 59.18% of the colority in HIM was removed.
3. After 90 min of the COP treatment, the BTPW toxicity was effectively reduced, as evidenced by the high incubation and low mortality rates. The GC-MS analysis showed that 38 types of organic pollutant species were completely removed, 8 were partially removed, and 7 were generated, which decreased the number of organic species from 45 to 15.

## Experimental

### Materials and reagents

BTPW used in this study was obtained from a pharmaceutical wastewater treatment plant located in Zhejiang Province, China. The treatment process for 2000 m^3^/d consists of hydrolysis acidification, A/A/O and a MBBR in sequence. All the collected wastewater was passed through a 0.45-μm filter, adjusted to pH = 3.1 ± 0.1 with hydrochloric acid, and stored at 4 °C before use. The main characteristics of the pretreated wastewater used in this study were as follows: 3.1 ± 0.1 pH_0_, 244 ± 3 mg/L of COD, 85 ± 2 mg/L of TOC, 224 ± 7 colority, 2050 ± 18 mg/L of chloride ions, 896 ± 7 mg/L of nitrate ions, 186 ± 4 mg/L of phosphate ions.

All chemical reagents were analytical reagent grade and used as received. Ultrapure water (≥18 MΩcm) purified by a Millipore Milli-Q Water system was used throughout this study. The details about the preparation of the MnO_2_ ceramsite as well as the methods and results of the characterizations and re-use performance are shown in the supporting information.

### Experimental procedure

To better explain the changes in the pharmaceutical EfOM during the COP, the SOP was also studied. SOP and COP were both carried out in a semi-continuous mode in a 1.2 L plexiglass reactor at room temperature by continuously bubbling a mixture of ozone/oxygen (inlet ozone concentration 20.25 ± 0.75 mg/L) through a microporous titanium plate at a flow rate of 400 mL/min. The reactor is a cylinder with a diameter of 80 mm and a height of 360 mm. Ozone was generated through an ozonizer (WH-H-Y, Wohuan Ozone Mechanical and Electrical Equipment Company, China) using dry oxygen as the feed gas. Before the experimental operation of the COP, 1 L of pretreated wastewater and 4 g of MnO_2_ ceramsite were first introduced into the reactor, and then, ozone was continuously fed into the reactor. Samples were withdrawn at 0, 10, 30, 50, 70 and 90 min, and the ozone was immediately quenched by sodium thiosulfate. After the ozone quenching, the samples were filtered by a 0.45-μm Teflon filter (Pall Corporation, USA) for analysis. After each run, the reactor was repeatedly washed with 20% hydrochloride acid and Milli-Q water. The device diagram for the experimental reaction is shown in Fig. S1. The SOP was carried out in an ozonation reactor under the same conditions without MnO_2_ ceramsite. Each process was performed in triplicate.

The toxicity assignment experimental steps were as follows. The organic matter was extracted from the water samples by Oasis HLB SPE cartridges, sequentially eluted with methanol and 1:1 n-hexane/acetone and then dissolved in DMSO after nitrogen blowing. The organic matter was diluted before use. Wild-type zebrafish were obtained from the Model Animal Research Center of Nanjing University. Embryos were obtained from 2 males and 1 female in a tank stimulated by the onset of light. The embryos were collected and placed at 28.5 °C in Petri dishes. Hours post fertilization (hpf) was used to represent the age of the embryos. Embryos at 4 hpf were randomly distributed in a 24-well plate at a density of 15 embryos/well at 28.5 °C with 2 mL of the diluted water samples. Dead embryos were removed daily to prevent necrotic effects. Each treatment was performed in quadruplicate.

### Analytical method

The ozone concentration in the gas phase was obtained by adopting an iodometric method^53^. The TOC and UV_254_ were analyzed by an Aurora 1030 W TOC analyzer (OI Analytical, USA) and a 2540 UV spectrophotometer (Shimadzu, Japan), respectively. The colority was measured by a colorimetic method according to the Chinese SEPA standard method^54^. The concentrations of humic acid, polysaccharides, and proteins were quantified by an improved Lowry method^55^, a colorimetric method^56^, and a bicinchoninic acid (BCA) kit method, respectively. Inorganic anions were determined by ion chromatography in a Dionex ICS-1000 apparatus equipped with an IonPac AS22 4 × 250 mm analytical column. The mobile phase was 4.5 mM Na_2_CO_3_ and 1.4 mM NaHCO_3_ with a flow rate of 1.2 mL/min at 30 °C. An XAD-8 macroporous adsorption resin was used to separate the hydrophilic and hydrophobic organic compounds in the water samples, according to Liu’s method^57^. The water samples were freeze-dried by a lyophilizer (Lacbconco, USA), and analyzed by a NEXUS 870 Fourier transform infrared spectrometer (NICOLET, USA). The distribution of the molecular weight was analyzed by high performance liquid chromatography-size exclusion chromatography (HPSEC) with a gel permeation column (Protein Pak 125, 7.8 × 300 mm, 10 μm, Waters)^58^. The compound identification was peformed by a 7890 A gas chromatograph (Agilent, USA) interfaced with a 5977B mass selective detector (Agilent, USA). The details of distribution of the molecular weight and GC-MS analysis procedure could be seen in supporting information. A mortality rate of 24 hpf and an incubation rate of 48 hpf were assessed using an SMZ745T dissecting microscope (Nikon, Japan).

### Statistical analysis

A statistical difference was evaluated using one-way analysis of variance (ANOVA). All analyses were performed by an SPSS statistical package (SPSS Inc., U.S.A.). A P value < 0.05 was accepted as significance and is marked with “*”.

### Data availability statement

The data within my uploaded manuscript file is available.

## Electronic supplementary material

supporting information
