# Supplementary material for: Insight into the characteristics, removal, and toxicity of effluent organic matter from a pharmaceutical wastewater treatment plant during catalytic ozonation
Source: Sci Rep. 2018 Jun 25;8:9581. doi: 10.1038/s41598-018-27921-0 (PMC6018431; doi:10.1038/s41598-018-27921-0)
Supplement: Supplementary file 1 — supporting information [file 41598_2018_27921_MOESM1_ESM.docx]

# Insight into the characteristics, removal, and toxicity of effluent organic matter from a pharmaceutical wastewater treatment plant during catalytic ozonation

Shuhan Wen, Lin Chen, Weiqi Li, Hongqiang Ren, Kan Li, Bing Wu, Haidong Hu, Ke Xu*

State Key Laboratory of Pollution Control and Resource Reuse

School of the Environment, Nanjing University

N.O.163, Xianlin Avenue, Qixia District, Nanjing 210023, Jiangsu, PR China

*Corresponding author:

Ke Xu

Tel.: 86-025-89680512; fax: 86-025-89680512

E-mail address: kexu@nju.edu.cn

# Preparation, characterization and re-use performance methods of catalysts

Attapulgite clay powder composed of 0.5~5 × 0.05~0.15 μm rod-shaped particles originating from Xuyi, Jiangsu, China, with attapulgite content of 99%, was used as [catalyst carrier](http://www.baidu.com/link?url=Z85nMFqGvmy50qEskO5zH_ytNJNrV80XeGlncAyk1KJT3SE9iwDMOA-lLIoa12kWdynvE1CGNVIs7FE7ItwB9FfCx0lgAAPjJum70vQeyhbU5WmCRBhWKmAQuTJW_uxw). MnO_2_ ceramsite was prepared by following steps. Mix attapulgite and commercial MnO_2_ powder (Macklin, China) well in the proportion of 5:1, then add water and mix the power make the mixture into pasty form. After 12 hours, shape the mixture into spheroidal particles and dry in the oven at the temperature of 105 °C, followed by calcining at 400 °C for 2 h, then cool in the ambient. Wash calcined spheroidal particles with 20% hydrochloride acid and water until the pH of wash solution is stable, and then stored in a dry vacuum oven for further use.

The crystalline structure of the MnO_2_ among MnO_2_ ceramsite was detected with an X-ray diffraction analyzer (RAPID II, Japan) by Mo radiation. The specific surface area of the catalyst was determined using the Brunauer-Emmet-Teller (BET) method by a surface area and pore distribution analyzer (ASAP 2020, USA). The point of zero charge (pH_PZC_) of catalysts were analyzed via potentiometer (Zetasizer Nano ZS90, United Kingdom). The surface morphology of catalyst was observed by scanning electron microscopy (Quanta 250 FEG, USA).

The re-use of MnO_2_ ceramsite was studied by the following experiment. The catalyst was washed with Milli-Q water until the pH of the washing solution remained unchanged. The catalyst was then placed in an oven at 60 °C for drying, and the same catalyst was reused 7 times. The oxalic acid solution (volume, 1 L; pH_0_, 3.1; mass concentration, 150 mg/L) and 4 g of MnO_2_ ceramsite were put into the reactor, followed by ozone-oxygen mixture. The inlet ozone concentration is 20.25 mg/L. Samples were withdrawn at 0, 5, 10, 20, and 30 min, and the ozone was immediately quenched by sodium thiosulfate. After the ozone quenching, the samples were filtered by a 0.45-μm Teflon filter for analysis. After each run, the reactor was repeatedly washed with 20% hydrochloride acid and Milli-Q water.

According to Chinese SEPA standard method [^1^](#_ENREF_1), the manganese ion content in the solution was determined by MKII & M6 atomic absorption spectrometry.

# Characterization and re-use performance of catalyst

Fig. S2 shows XRD patterns of commercial MnO_2_ powder, calcined commercial MnO_2_ powder, ceramsite and MnO_2_ ceramsite. Raw commercial MnO_2_ consisted of type γ and α MnO_2_. After calcination, γ-MnO_2_ was converted to β-MnO_2_, while α-MnO_2_ could be still detected. The transition of the MnO_2_ crystal form is similar to the research by Ren [^2^](#_ENREF_2). Fig. S2 (c) identifies that α-MnO_2_ and β-MnO_2_ succeeded in loading in ceramsite. The same components were used in COPs[^3-6^](#_ENREF_3) as catalytically active substance.

The surface morphology of MnO_2_ powder and the MnO_2_ ceramsite catalyst is shown in Fig. S3. Attapulgite has fibrous or felt-like structure, and spherical MnO_2_ particles [^7^](#_ENREF_7) were distributed on the surface of attapulgite ceramsite. Such a surface structure of MnO_2_ ceramsite had a larger specific surface area, which could provide more active sites for ozone and hydroxyl radicals to improve the removal efficiency of organic matters.

The physical properties of MnO_2_ ceramsite and ceramsite are summarized in Table S1. The surface area of MnO_2_ ceramsite is 109.63 m^2^/g. The total pore volume and average pore diameter was 0.32 cm^3^/g and 11.59 nm, respectively. Compared with the ceramic, the surface area of MnO_2_ ceramic increased slightly, while the total pore volume and average pore diameter decreased lightly. MnO_2_ ceramic had a low pH_PZC_ value of 1.0, which is 0.2 higher than that of ceramic.

As can be seen from Fig. S4, when the same catalysts were reused seven times, the removal rate of oxalic acid remained at 61.01 ~ 67.13%, indicating that the MnO_2_ ceramsite has good performance in catalyst re-use.

It can be seen from Table S2 that the Mn^2+^ dissolution amount is very small, and the dissolution value is 0.01 ~ 0.02 mg/L during 7 runs of using, which is much lower than the Mn^2+^ content of A grade standard (2 mg/L) in the wastewater quality standards for discharge to municipal sewers (CJ 343-2010)[^8^](#_ENREF_8), so Mn^2+^ pollution caused by using MnO_2_ ceramsite could be ignored.

# GC-MS analysis procedure

Before GC-MS analysis, the pretreatment steps of the water sample are as follows. Uniformly mix 250 mL water sample, 2 g NaCl and 60 mL methylene chloride, then shake well and let stand for 10 min till the organic phase to separate and filter. After the filtrate was concentrated to dryness in a 45 °C water bath, the volume was adjusted to 1 mL.

The parameters of purge-and-trap parameter, gas chromatography, and mass selective detector are as follows:

**Purge-and-trap (OI, 4660) parameter:** purge temperature: room temperature, purge rate: 40 mL/min, purge time: 10 min, dry purge time: 1 min, pre-desorption temperature: 180 °C, desorption temperature: 190 °C, desorption time: 2 min, bakeout time: 6 min

**Gas chromatography parameter (Agilent 7890A**)**:** injector temperature: 220 °C, injector type: spitless (10:1), temperature programming: 35 °C (2 min) → 5 °C/min → 120 °C → 10 °C/min → 220 °C (2 min), carrier gas: helium, gas flow rate: 1.0 mL/min

**Mass selective detector parameter (Agilent** **5977B):** ion source: EI source, ion source temperature: 230 °C, ion energy: 70 eV, scanning mode: full scan, scanning range: m/z35-270amu, solvents delay: 2.0 min, electron multiplying voltage: same with tune voltage, transfer line temperature: 280 °C

# The molecular weight distribution analysis procedure and the details of exclusion time

## Molecular weight distribution analysis procedure

The distribution of the molecular weight was analyzed by a high performance liquid chromatography-size exclusion chromatography (HPSEC) with a series-connected Ultraviolet (UV) and used a gel permeation column (Protein Pak 125, 7.8 × 300 mm, 10 μm, Waters) according to Wang’s method [^9^](#_ENREF_9). Before analysis for distribution of the molecular weight, the samples were filtered by a 0.22-μm Teflon filter (Pall Corporation, USA). A molecular weight of 210, 3200, 17,000, 32,000, 77000 Da of sodium polystyrene sulfonates (PSS, Fluka) was used as the standards to detect at 254 nm. The calibration curve was established by plotting the logarithm of molecular weight (logMw) versus the retention time and could be seen in Fig. S5. The mobile phase was composed of phosphate buffer solution (1.6 mmol/L NaH_2_PO_4_ + 2.4 mmol/L Na_2_HPO_4_) and 0.025 mol/L Na_2_SO_4_ aqueous solution at a flow rate of 1.0 mL/min. The sample volume was 20 μL.

## The details of exclusion time (retention time)

The exclusion time of this paper could be seen in Fig. 5. The retention time of untreated HIM in BTPW from left to right in Figure 5(a) is 7.40 min, 7.86 min, 8.68 min, 9.45 min and 13.85 min, respectively. The retention time of HIM in BTPW treated with SOP from left to right in Figure 5(a) is 7.38 min, 7.81 min, 8.46 min, 9.41 min and 12.75 min, respectively. The retention time of HIM in BTPW treated with COP from left to right in Figure 5(a) is 7.38 min, 7.80 min, 8.45 min and 9.40 min, respectively. The retention time of untreated HOM in BTPW from left to right in Figure 5(b) is 7.46 min, 8.08 min, 8.77 min, 9.22 min, and 13.01 min, respectively. The retention time of HOM in BTPW treated with SOP from left to right in Figure 5(b) is 7.41 min, 7.88 min, 8.68 min, 9.23 min and 10.47 min, respectively. The retention time of HOM in BTPW treated with COP from left to right in Figure 5(b) is 7.45 min, 7.88 min, 8.66 min, 9.22 min and 10.50 min, respectively.

# The study about the generation of ·OH and the pollutants adsorption caused by MnO_2_ ceramsite

## Experimental procedure

Adsorption of oxalic acid by MnO_2_ ceramics: The oxalic acid solution (volume, 1 L; pH_0_, 3.1; mass concentration, 150 mg/L; the same below) and 4 g of MnO_2_ ceramsite were first introduced into the reactor, and then, oxygen with a purity of 99% (400 mL/min) was continuously fed into the reactor. Samples were withdrawn at 0, 5, 10, 20, and 30 min, and the samples were filtered by a 0.45-μm Teflon filter for analysis. After each run, the reactor was repeatedly washed with 20% hydrochloride acid and Milli-Q water (the same below).

Oxidative degradation of oxalic acid by solo ozone: The mixture of ozone-oxygen gas was introduced into the oxalic acid solution. The inlet ozone concentration is 20.25 mg/L. Samples were withdrawn at 0, 5, 10, 20, and 30 min, and the ozone was immediately quenched by sodium thiosulfate. After the ozone quenching, the samples were filtered by a 0.45-μm Teflon filter for analysis.

Oxidative degradation of oxalic acid by MnO_2_ ceramsite: The oxalic acid solution and 4 g of MnO_2_ ceramsite were put into the reactor, followed by ozone-oxygen mixture. The inlet ozone concentration is 20.25 mg/L. The following experimental steps were the same as above.

Oxidative degradation of oxalic acid by MnO_2_ ceramsite in presence of Tert-butyl alcohol (TBA): The oxalic acid solution, 4 g of MnO_2_ ceramsite and 1 mL TBA were put into the reactor, followed by ozone-oxygen mixture. The inlet ozone concentration is 20.25 mg/L. The following experimental steps were the same as above.

The LC1200 high performance liquid chromatography (Agilent, USA) was used to determine the concentration of oxalic acid. The analysis conditions were as follows: the column, an Agilent SB-C18 column (250 mm × 4.6 mm); UV detection wavelength, 210 nm; the mobile phase, mixed solution of V (methanol): V ( pH = 2.7 phosphate buffer solution) = 15: 85; flow rate, 1.0 mL/min; column temperature, 25 °C; sample volume, 10 μL; retention time, 2.6 min.

Each process was performed in triplicate.

## Results and discussion

The degradation efficiency of oxalic acid by different oxidation processes could be seen in Fig. S6. When oxalic acid concentration, solution pH and inlet ozone concentration were 150 mg/L, 3.1 and 20.25 mg/L, respectively, after 30 min the removal rate of oxalic acid was 10.69%. Under the same reaction conditions, added 4 g of MnO_2_ ceramsite, the removal rate of oxalic acid was 66.99%, which was 6.3 times that of solo ozone. In addition, only 8.26% of oxalic acid was removed by MnO_2_ ceramsite adsorption. It could be seen that the removal efficiency of oxalic acid by catalytic ozonation is much greater than that of the sum of sole ozone and MnO_2_ ceramsite adsorption. The reaction rate constant of oxalic acid with ·OH is 5.3 × 10^6^ L/(mol·s) [^10^](#_ENREF_10), which is much greater than that with ozone. Therefore, it is speculated that MnO_2_ ceramsite catalyzes ozone to generate more hydroxyl radicals and increases the removal rate of oxalic acid.

TBA is a typical hydroxyl radical inhibitor whose the reaction rate constant with hydroxyl radical is 5×10^8^ L/(mol·s), so it can quickly consume hydroxyl radicals[^11^](#_ENREF_11). At the same time, TBA hardly reacts with ozone molecules[^12^](#_ENREF_12). Hence, when adding TBA during the experiment, the removal rate of oxalic acid is significantly reduced could indicate that the reaction mainly follows the hydroxyl radical reaction mechanism.

The experimental results showed that the removal rate of oxalic acid was significantly reduced due to the addition of TBA. The removal rate of oxalic acid decreased from 66.99% to 38.16%, indicating that 28.83% of oxalic acid was removed by hydroxyl radicals during COP. The amount of oxalic acid removed by the oxidation of hydroxyl radicals accounted for 43.04% of the total removal. It can be indicated that MnO_2_ ceramsite could catalyze ozone to generate more hydroxyl radicals.

# Tables and figures


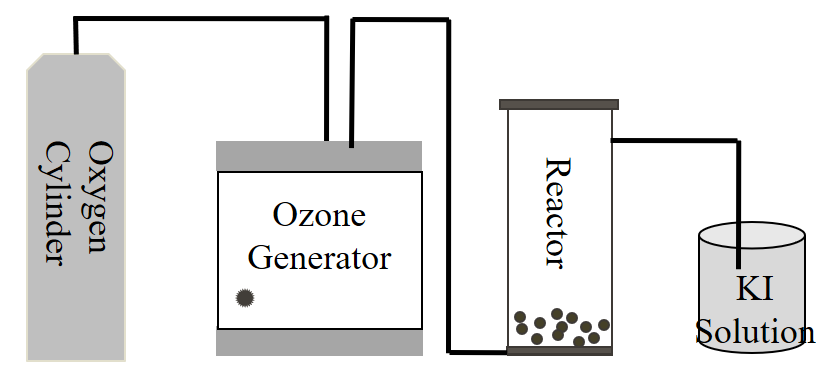


Fig. S1 The device diagram of experimental reaction


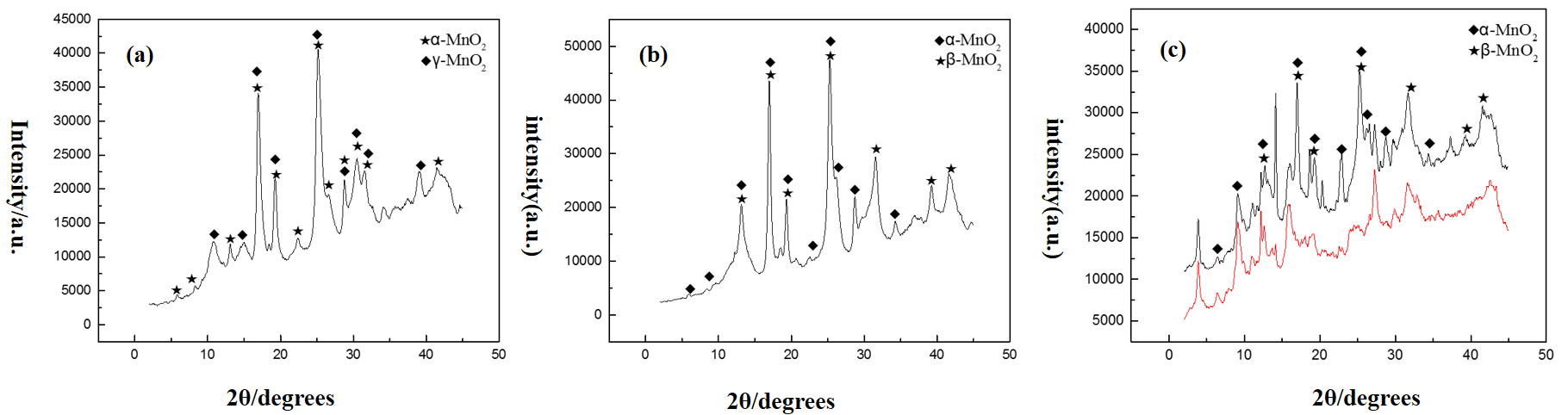


Fig. S2 XRD patterns: (a) commercial MnO_2_ powder, (b) commercial MnO_2_ powder calcined at 400 °C for two hours, (c) ceramsite and MnO_2_ ceramsite, red solid line for ceramsite, and black solid line for MnO_2_ ceramsite


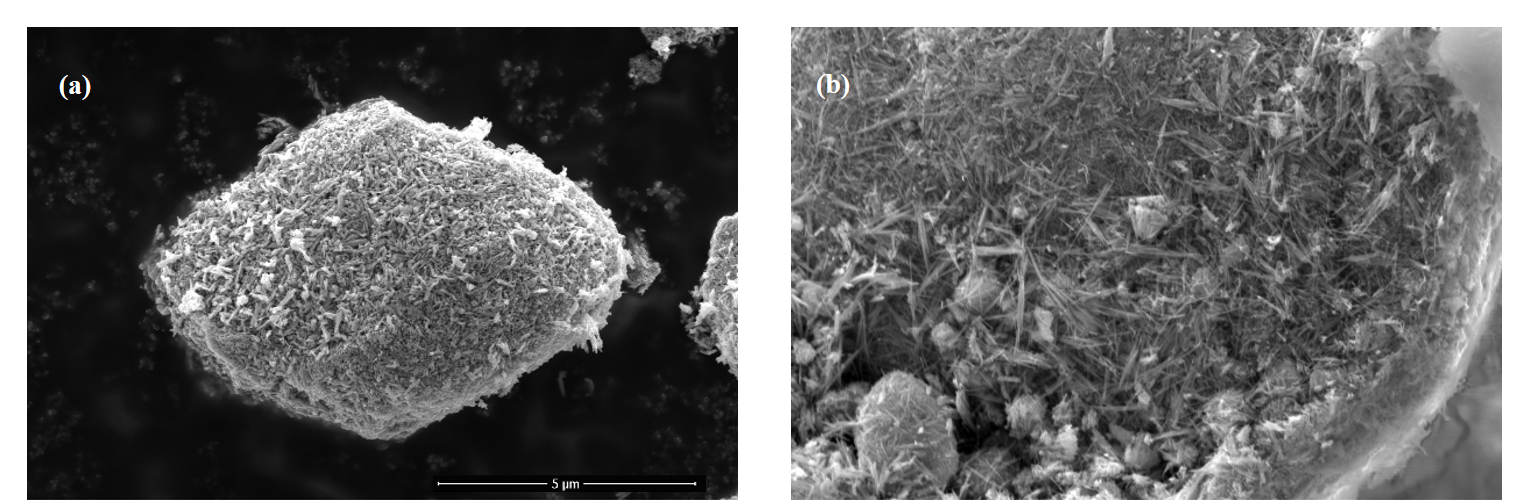


Fig. S3 SEM images of calcined MnO_2_ powder (a) and MnO_2_ ceramsite (b)


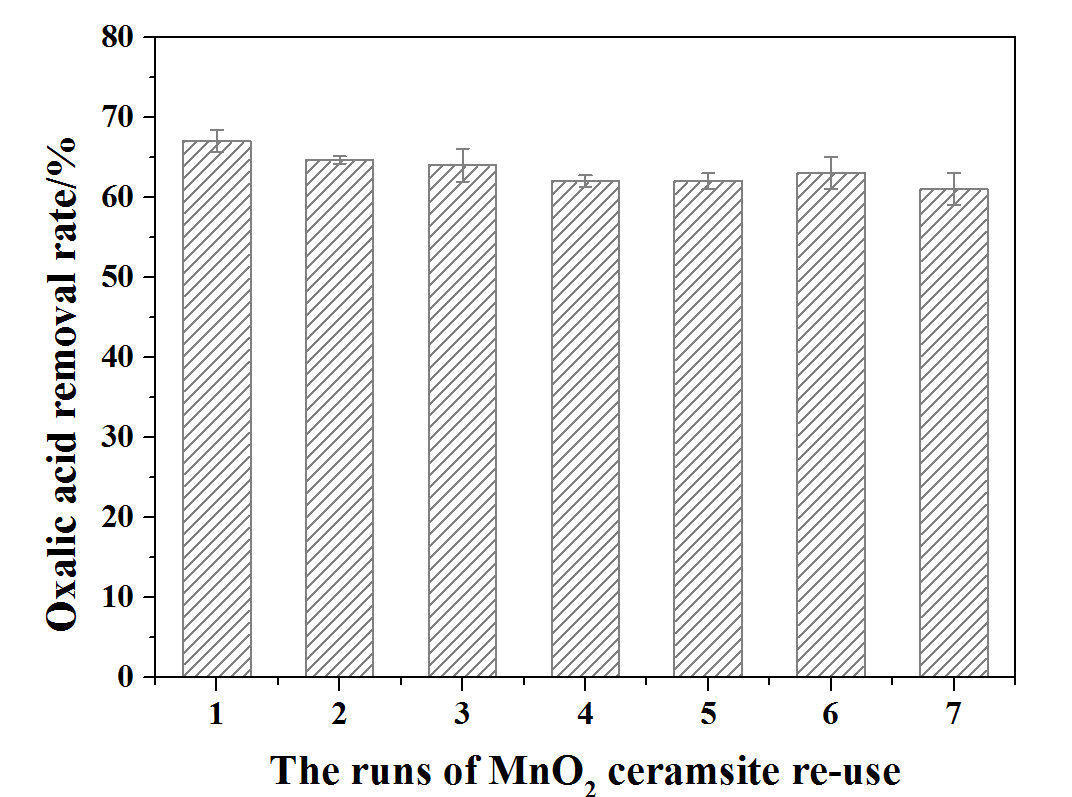


Fig. S4 The removal rate of oxalic acid by catalytic ozonation in presence of MnO_2_ ceramsite under the number of different catalyst re-use (experimental conditions: oxalic acid concentration, 150 mg/L; pH_0_, 3.11; catalyst dosage, 4.0 g/L; gas flow, 400 mL/min; inlet ozone concentration, 20.25 mg/L; reaction volume, 1.0 L; reaction time, 30 min)


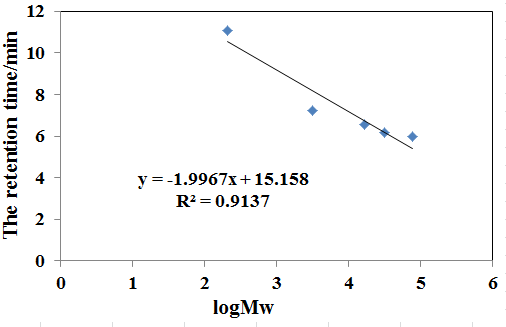


Fig. S5 The calibration curve for molecular weight (logMw) and the retention time


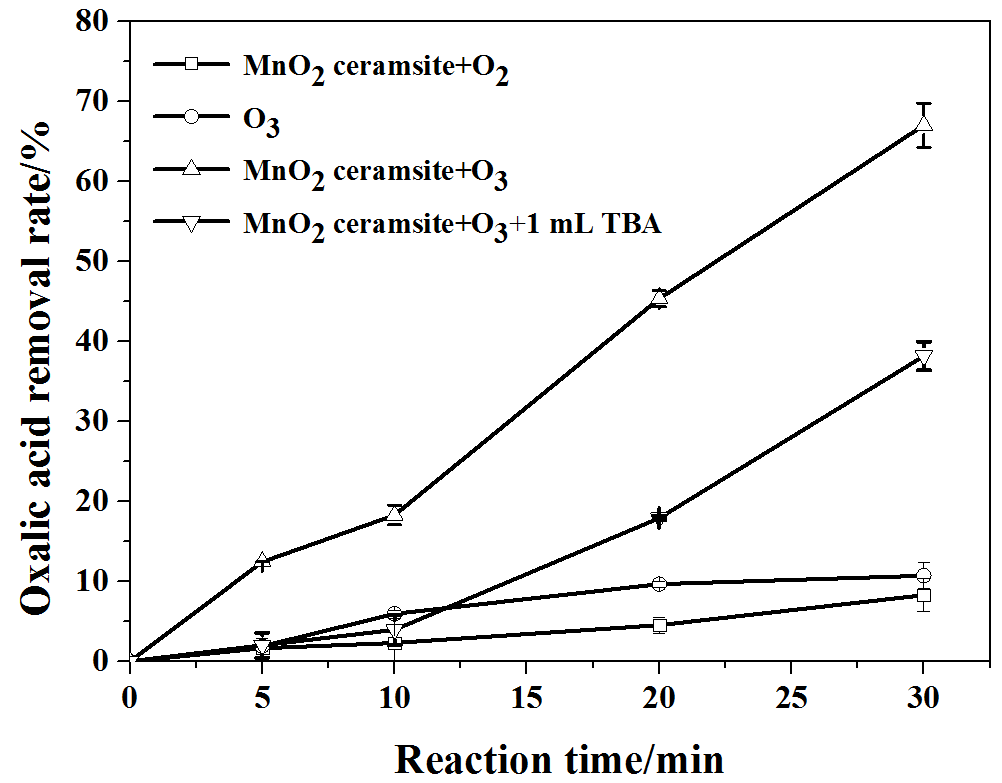


Fig. S6 The degradation efficiency of oxalic acid by different oxidation processes (experimental conditions: oxalic acid concentration, 150 mg/L; pH_0_, 3.11; the inlet ozone concentration, 20.25 mg/L; reaction volume, 1.0 L; reaction time, 30 min)

**Table S1 Physical properties of MnO_2_ ceramsite and ceramsite**

| **Samples** | **BET surface area（m^2^/g）** | **Total pore volume（cm^3^/g）** | **Average pore diameter（nm）** | **pH_PZC_** |
| --- | --- | --- | --- | --- |
| MnO_2_ ceramsite | 109.63 | 0.32 | 11.59 | 1.0 |
| Ceramsite | 100.39 | 0.35 | 13.96 | 0.8 |

**Table S2 The dissolution content of Mn^2+^ during catalytic ozonation in presence of reused MnO2 ceramsite**

| **The runs of MnO2 ceramsite re-use** | **1** | **2** | **3** | **4** | **5** | **6** | **7** |
| --- | --- | --- | --- | --- | --- | --- | --- |
| Dissolution amount of Mn^2+^/mg/L | 0.01 | 0.02 | 0.02 | 0.01 | 0.01 | 0.01 | 0.01 |

# References

1 China, E. o. Water and Wastewater Monitoring Analysis Method(fourth ed.). *Chinese Environment Science Publisher*, (Beijing (2002)).

2 Ren, T. Z., Yuan, Z. Y., Du, G. H. & Su, B. L. Facile preparation of nanostructured manganese oxides by hydrotreatment of commercial particles. *Stud. Surf. Sci. Catal.* **162**, 425-432, (2006).

3 Dong, Y. M., Yang, H. X., He, K., Song, S. Q. & Zhang, A. M. *β*-MnO_2_ nanowires: a novel ozonation catalyst for water treatment. *Appl. Catal. B-Environ.* **85**, 155-161, (2009).

4 Nawaz, F. *et al.* Catalytic ozonation of 4-nitrophenol over an mesoporous *α*-MnO_2_ with resistance to leaching. *Catal. Today* **258**, 595-601, (2015).

5 Li, G. *et al.* Efficient catalytic ozonation of bisphenol-A over reduced graphene oxide modified sea urchin-like *α*-MnO_2_ architectures. *J. Hazard. Mater.* **294**, 201-208, (2015).

6 Saputra, E. *et al.* Different crystallographic one-dimensional MnO_2_ nanomaterials and their superior performance in catalytic phenol degradation. *Environ. Sci. Technol.* **47**, 5882-5887, (2013).

7 Chen, S., Duan, J. J., Han, W. & Qiao, S. Z. A graphene-MnO_2_ framework as a new generation of three-dimensional oxygen evolution promoter. *Chem. Commun.* **50**, 207-209, (2014).

8 Beijing Municipal Engineering Management Office & Office, S. D. A. *Wastewater quality standards for discharge to municipal sewers*. (China Standard Press, 1987).

9 Wang, D. *et al.* Chromium speciation in tannery effluent after alkaline precipitation: Isolation and characterization. *J. Hazard. Mater.* **316**, 169-177, (2016).

10 Sehested, K., Getoff, N., Schwoerer, F., Markovic, V. M. & Nielsen, S. O. Pulse radiolysis of oxalic acid and oxalates. *Journal of Physical Chemistry* **75**, 749-755, (1971).

11 Andreozzi, R., Caprio, V., Insola, A. & Marotta, R. Advanced oxidation processes (AOP) for water purification and recovery. *Catal. Today* **53**, 51-59, (1999).

12 Gurol, M. D. & Akata, A. Kinetics of ozone photolysis in aqueous solution. *AIChE Journal* **42**, 3283-3292, (1996).
